# *In Vivo* Cardiotoxicity Induced by Sodium Aescinate in Zebrafish Larvae

**DOI:** 10.3390/molecules21030190

**Published:** 2016-02-23

**Authors:** Jinfeng Liang, Wangdong Jin, Hongwen Li, Hongcui Liu, Yanfeng Huang, Xiaowen Shan, Chunqi Li, Letian Shan, Thomas Efferth

**Affiliations:** 1 Zhejiang Center for Drugs and Cosmetics Evaluation, Zhejiang Province Food and Drug Administration, Hangzhou 310012, China; lvminella@hotmail.com; 2 Institute of Orthopaedics and Traumatology, Zhejiang Chinese Medical University, No.548 Binwen Road, Binjiang District, Hangzhou 310053, China; wangdong.jin@hotmail.com; 3 Experimental and Training Center, Zhejiang Pharmaceutical College, Ningbo 315100, China; hongwen_lee@sohu.com; 4 Hunter Biotechnology, Inc., Transfarland, Hangzhou 310012, China; lhc@zhunter.com (H.L.); 10710@etransfar.com (Y.H.); 5 Zhejiang Provincial Key Lab for Technology and Application of Model Organisms, Wenzhou Medical University, Wenzhou 325035, China; 6 Department of Pharmaceutical Biology, Institute of Pharmacy and Biochemistry, Johannes Gutenberg University, Mainz 55128, Germany; efferth@uni-mainz.de

**Keywords:** sodium aescinate, zebrafish, larvae, cardiotoxicity, MNLC, LC_10_

## Abstract

Sodium aescinate (SA) is a widely-applied triterpene saponin product derived from horse chestnut seeds, possessing vasoactive and organ-protective activities with oral or injection administration in the clinic. To date, no toxicity or adverse events in SA have been reported, by using routine models (*in vivo* or *in vitro*), which are insufficient to predict all aspects of its pharmacological and toxicological actions. In this study, taking advantage of transparent zebrafish larvae (*Danio rerio*), we evaluated cardiovascular toxicity of SA at doses of 1/10 MNLC, 1/3 MNLC, MNLC and LC_10_ by yolk sac microinjection. The qualitative and quantitative cardiotoxicity in zebrafish was assessed at 48 h post-SA treatment, using specific phenotypic endpoints: heart rate, heart rhythm, heart malformation, pericardial edema, circulation abnormalities, thrombosis and hemorrhage. The results showed that SA at 1/10 MNLC and above doses could induce obvious cardiac and pericardial malformations, whilst 1/3 MNLC and above doses could induce significant cardiac malfunctions (heart rate and circulation decrease/absence), as compared to untreated or vehicle-treated control groups. Such cardiotoxic manifestations occurred in more than 50% to 100% of all zebrafish treated with SA at MNLC and LC_10_. Our findings have uncovered the potential cardiotoxicity of SA for the first time, suggesting more attention to the risk of its clinical application. Such a time- and cost-saving zebrafish cardiotoxicity assay is very valid and reliable for rapid prediction of compound toxicity during drug research and development.

## 1. Introduction

*Aesculus hippocastanum* (Hippocastanaceae), the worldwide-distributed horse chestnut with excellent resistance to environmental conditions, possesses therapeutic properties of relieving tissue edema, recovering vasopermeability and eliminating pressure caused by edema [1,2]. Sodium aescinate (SA) is a triterpene saponin derived from horse chestnut seeds, widely used as a dietary supplement and skin care product, as well as a pharmaceutical. As a bioactive natural product, SA has recently been used in clinical therapy for its anti-apoptotic, anti-edematous, anti-inflammatory and antioxidative effects [3,4,5]. Oral tablets, injections and topical gel are the common formulations of SA in the clinic, for the treatments of hemorrhoids, chronic venous insufficiency and encephaledema or post-traumatic/surgery edema [6]. The mechanism of its actions includes the increase of intravenous tension, improvement of microcirculation, attenuation of exudative inflammation response, enhancement of immunity functions, decrease of oxidative injury, protection of endothelial cells from hypoxia and inflammation injury and inhibition of phosphate, histamine and bradykinin release [7,8,9,10]. A number of preclinical and clinical research works have supported the above findings. However, to date, no report has concerned the side effect or toxic profile of SA, which is necessary for understanding of the pharmacologic action of SA in the clinic.

Zebrafish (*Danio rerio*), the cyprinid schooling teleost, is small for a vertebrate, reaching only 3 cm in length. During its embryonic and larval stages, zebrafish is only 2–3 mm long, which can live for days in individual wells of standard plates (96 or 384 wells/plate) with conventional nutrients [11]. It possesses orthologs for >70% of human genes and >86% of 1318 human drug targets [12,13]. Genes identified in the original zebrafish organogenesis screens have been consistently validated in humans or mice [14,15]. In many cases, zebrafish and human gene discovery have occurred side by side [16,17]. The observed positive or adverse effects in zebrafish and human exposed to the same chemicals are strongly correlated [18,19], indicating their remarkable pharmacologic homologies and a high representativity of zebrafish for human in the experimental research field. As zebrafish becomes more widely used, it has been a popular and ideal model organism for studying genetics, developmental biology, pharmacology, DNA damage repair, cancer and other diseases, especially for studying the toxic effects of chemicals or drugs *in vivo* [20,21].

Organ-specific toxicity remains the most common reason for the failure of drug therapy or for drug withdrawal from the market [11]. Fast and precise estimation of such toxicity risk is thereby of importance for drug use, which can be implemented by zebrafish. In this study, to reveal the toxic profile and *in vivo* adverse effect of SA, we tested the cardiotoxicity of this component by using transparent zebrafish larva. This could be the first report on the toxicity of SA.

## 2. Results

### 2.1. MNLC and LC_10_

**Figure 1:**
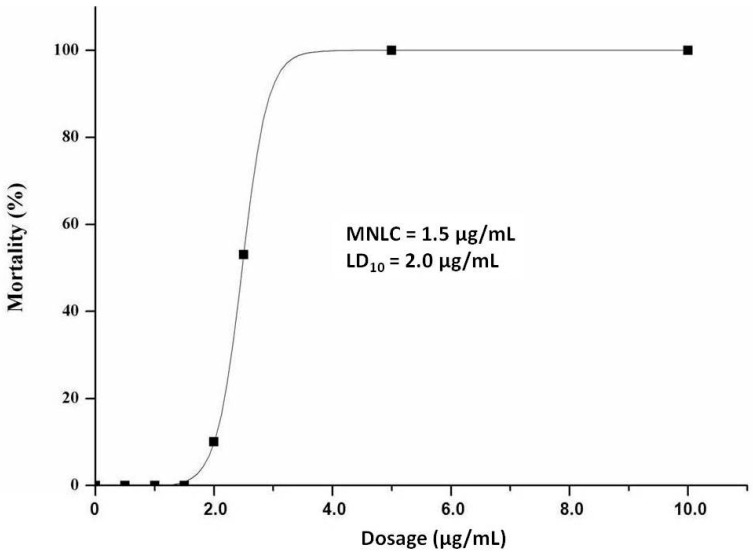
Sodium aescinate (SA)-induced dose-dependent zebrafish larvae mortality between 72 and 120 h post-fertilization (hpf) by yolk sac microinjection (*n* = 30 zebrafish per treatment; SA solution volume was 10 nL).

The SA-induced zebrafish lethality curve is presented and a dose-dependent mortality found in Figure 1. No lethal effect was observed within SA doses from 0.1 to 1.5 μg/mL, while no zebrafish survived with SA treatment at doses above 5.0 μg/mL. MNLC and LC_10_ of SA were thereby estimated as 1.5 and 2.0 μg/mL, using sigmoidal regression in Origin 8.0 software. During the double-blind observation, no observed adverse effect level (NOAEL) of SA could be estimated as 0.1 μg/mL. Thus, 1/10 of MNLC (0.15 μg/mL), 1/3 of MNLC (0.5 μg/mL), MNLC (1.5 μg/mL) and LC_10_ (2.0 μg/mL) were adopted for cardiotoxicity assessment.

### 2.2. Visual Assessment of Cardiotoxicity

**Figure 2:**
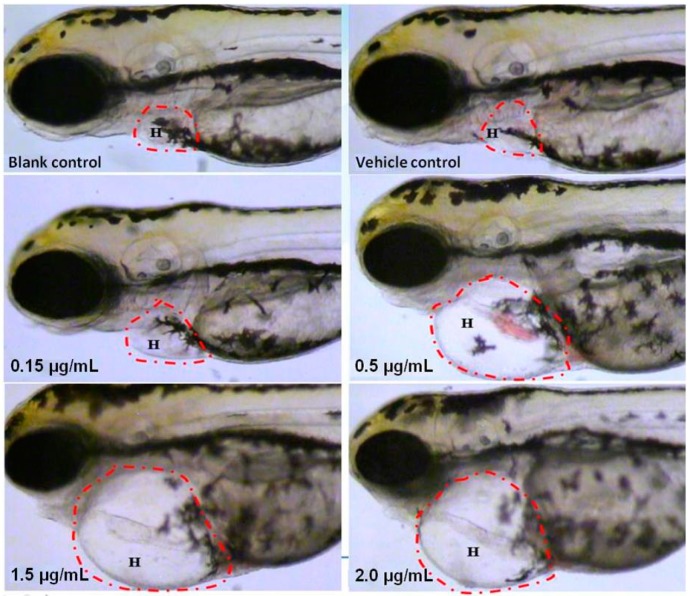
Visual observation of zebrafish larvae at 120 hpf after microinjection of SA at 72 hpf. The circled area (H) is zebrafish heart and pericardium.

Cardiovascular toxicity-associated morphological abnormalities were observed and are shown in Figure 2. In both untreated and vehicle control groups, no morphological changes were found in the heart and pericardium of zebrafish. However, with increasing doses of SA treatment, increasingly severe manifestations of cardiac and pericardial abnormalities, such as heart malformations, cardiac looping defects and pericardial edema, were observed in a dose-dependent manner. Especially in groups with 1.5 μg/mL and 2.0 μg/mL of SA, the zebrafish pericardium was enlarged, edematous and filled with turbid fluid, which appeared about four-times larger than that of control groups. Moreover, SA at 0.15 μg/mL caused minimal, but significant abnormality in the zebrafish heart, indicating this dosage as the lowest observed adverse effect level (LOAEL).

### 2.3. Quantitative Observation of Cardiotoxicity

During the visual assessment, incidences of heart malformations, pericardial edema, circulation abnormalities, thrombosis and hemorrhage were observed and quantitatively assessed. As illustrated in Table 1, untreated and vehicle-treated zebrafish showed no abnormality in the heart and pericardium, whereas AS-treated ones exhibited typical heart malformations, pericardial edema and circulation decrease and absence in a dose-dependent manner. At doses of MNLC and LC_10_, all treated zebrafish showed heart malformations and pericardial edema (100% of incidences), and almost more than half of the zebrafish showed circulation abnormalities, indicating severe cardiovascular damage induced by SA. No thrombosis or hemorrhage was found from any treatment.

**Table 1:** Incidences (%) of visual cardiotoxic manifestations in zebrafish larvae.

Manifestations	Blank Control	Vehicle Control	SA (μg/mL)			
			0.15	0.5	1.5	2.0
Heart malformations	0	0	10.0	70.0	100.0	100.0 *
Pericardial edema	0	0	10.0	70.0	100.0	100.0 *
Circulation decrease	0	0	3.3	36.7	53.3	66.7 *
Circulation absence	0	0	0	3.3	46.7	100.0 *
Thrombosis	0	0	0	0	0	0
Hemorrhage	0	0	0	0	0	0

* Four of 30 zebrafish were dead, and 26 zebrafish were actually observed.

### 2.4. Heart Rate and Heart Rhythm

**Figure 3:**
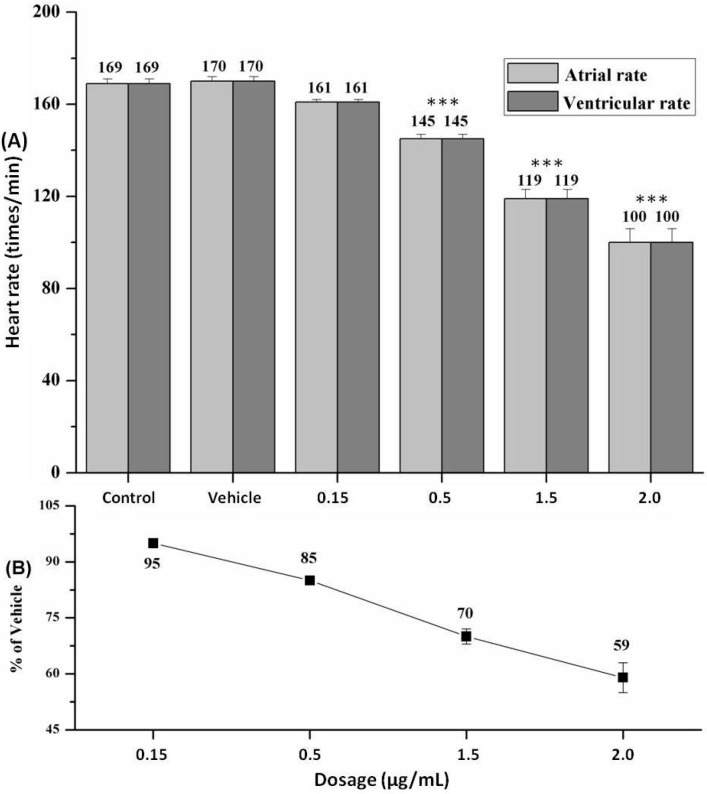
Zebrafish heart rate changes. (**A**) Heart rate (times/minute); (**B**) relative heart rate (%) of SA-treated zebrafish. All data are represented as the mean ± standard deviation (SD). *** *p* < 0.001 *vs.* vehicle control.

As illustrated in Figure 3, heart rate changes were found in a dose-dependent manner with SA treatment and a statistically-significant decrease caused by SA at 1/3 MNLC and above doses as compared to the vehicle control (all *p* < 0.001). Within 48 h of treatment, SA at the LC_10_ dose induced approximately a 50% decrease of heart rate, exhibiting an acute and strong cardiotoxicity. For heart rhythm assessment, no difference was found between the atrial rate and ventricular rate.

## 3. Discussion

**Figure 4:**
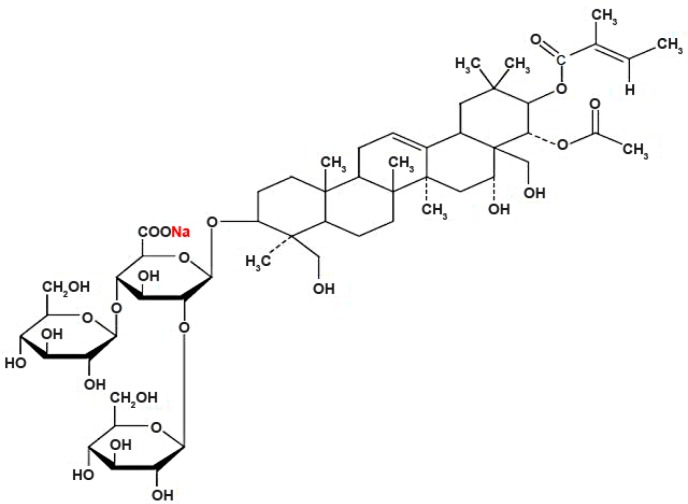
Chemical structure of sodium aescinate (SA).

As a major active compound in the horse chestnut seed, SA (C_55_H_85_NaO_24_; Figure 4) has been widely applied in the clinic, possessing vasoactive and organ (cerebral cortex, lung and liver) -protective activities in the clinic [4,5,6,22]. There is no report to date on the adverse event or toxic effect of SA. Additionally, in many cases, SA exerts functions through eliminating cell edema, ameliorating microcirculation and scavenging toxic free radicals [23,24,25]. Through the animal experiments, all available publications have confirmed such a “good face” of SA. However, unimaginably, but indeed, SA has another unbeknown face termed the “evil face”, which is of cardiovascular toxicity revealed by the present study via zebrafish larvae.

Routinely, *in vitro* cellular systems and *in vivo* rodent models are used for determining the side effects of a drug on an organ or the whole body. However, although the *in vitro* systems enable high-throughput testing of cytotoxicity and the *in vivo* models exhibit high similarity with human diseases, the prediction of drug toxicity remains extremely difficult due to the disadvantages of such traditional methods [26]. For instance, the *in vitro* systems fail to recapitulate the complexity of the intact organism, whilst the *in vivo* models are low throughput, expensive and time consuming [27]. In contrast, zebrafish take comparative advantages over the traditional experimental animals and cell lines: (1) short experimental period owing to its large-scale generation and rapid organogenesis and development; (2) high-throughput application requiring only a simple and inexpensive breeding technique; (3) real-time visible observation of cardiovascular response, since larval zebrafish are transparent; (4) remarkable pharmacological homologies with humans [11,28,29,30,31,32,33]. More importantly, zebrafish are extremely sensitive to the toxic stimulus and begin dying earlier, being suitable for early prediction of drug toxicity. Several studies have confirmed that zebrafish are a highly predictive animal model for *in vivo* screening of cardiovascular toxicity [34,35,36,37]. The transparency of zebrafish larvae indeed facilitates rapid evaluation of the heart rate, heart rhythm and pericardial edema [33]. Utilizing only simple heart-rate responses of zebrafish, investigators can establish an excellent correlation with known adult human cardiac toxicity and recapitulate clinical relevant results [31]. Previously, the overall prediction success rate of zebrafish for cardiotoxic and non-cardiotoxic drugs attained 100% [34], which has been ranked as excellent (>85%) for identifying cardiovascular toxicity agents by the ECVAM (European Center for the Validation of Alternative Methods) criteria [38]. In sum, all of the above evidence supports the use of zebrafish as a predictive animal model for rapid screening of *in vivo* cardiovascular toxicity.

In the present study, the larval zebrafish cardiotoxicity assay was performed to disclose the unbeknown “evil face” of the widely-applied vasoactive agent, SA, followed by visible observations of the morphology and motion of heart and pericardium, as well as the quantitative analysis of cardiac function. As demonstrated by our results, within 48 h of treatment, SA at 1/10 MNLC and above doses could induce obvious cardiac and pericardial malformations (Figure 2), whilst at 1/3 MNLC and above doses, it could induce significant cardiac malfunctions (heart rate and circulation decrease) (Figure 3). Such cardiotoxic manifestations occurred in more than 50% to 100% of the zebrafish with SA treatment at doses of MNLC and LC_10_ (Table 1). In all of the experiments, no death of zebrafish was found in untreated and vehicle-treated groups and also no obvious difference in assessed endpoints was found between two control groups. In addition, the microinjection route used for SA can control the exact dose given to zebrafish that could be easily converted to the dose in humans or mammals if a conversion factor could be validated, which is superior and more valid than other administrations [34]. Nevertheless, a validated dose conversion factor from zebrafish to human is to date unavailable, so that the clinical implication of our findings for SA safety considering its marketed dosage could only be extrapolated based on the reported studies. For instance, Ducharme *et al*. demonstrated a significant positive correlation of lethality values (50% lethal concentration/dose, LC_50_/LD_50_) and toxicity endpoints between zebrafish and rodents (mouse and rat) [39], while Zhang *et al*. conducted a comparison of toxicity between zebrafish and mammalian models and found the similar LC_50_ value of cardiotoxic compound (e.g., epirubicin) in both zebrafish and mouse [40]. Under this circumstance, the LOAEL of SA (0.15 μg/mL) in our study could be converted as a potentially cardiotoxic dosage (0.15 mg/kg) to mouse (i.v.). By converting the SA’s marketed dosage from human (0.1 to 0.4 mg/kg) to mouse (~0.9 to 3.6 mg/kg), it is found that the minimal clinical dosage (0.9 mg/kg for mouse) was higher than the potentially cardiotoxic dosage (0.15 mg/kg for mouse), indicating a potential hazard of SA to the consumers. Together, our findings indicate that the zebrafish cardiotoxicity assay is very valid and reliable, and for the first time, the potential toxicity of SA to the human cardiovascular system can thereby be confirmed.

As a predictive vertebrate animal model, zebrafish larvae are apparently able to capture toxicity with toxic metabolites that are unlikely to be found *in vitro* [41]. Many studies have confirmed that the toxicity profiles of zebrafish and mammals are strikingly similar [33,34,41]. This animal can thereby be used to rapidly predict the toxicity of compounds in the early stages of drug development, reduce drug failure at later stages of drug development, prioritize compounds for further preclinical and clinical studies and, more importantly, refilter out potentially unsafe subjects from commonly-used drugs on the market, such as SA, before they cause harm to humans [34,41]. Thus, the time- and cost-saving, sensitive, effective and high throughput characteristics of the cardiotoxicity assay using zebrafish larvae is clinically significant to public health in the drug screening and application field, especially when the routine methods fail to work under some circumstances. Without the help of zebrafish in this study, the "evil face" of SA may never have been discovered. Here, we suggest that doctors and patients should be more cautious with SA treatment, and the SA-treated patients should have a periodic inspection of the cardiovascular system. Our further studies are in progress to explore SA’s toxic mechanism in zebrafish and to reassess its cardiotoxicity in other model systems, which can provide more valuable evidence for the SA-associated health risk.

## 4. Materials and Methods

### 4.1. Chemical Preparation

SA (Batch Number 1403137) in powder was provided by Zhejiang Reviewer Center of Pharmaceutical and Cosmetic Products (Hangzhou, China) and stored at room temperature. It was dissolved with 0.9% NaCl solution for use.

### 4.2. Zebrafish Handling

A wild-type AB strain of zebrafish (Batch Number 20150319) was used in this study. Adult zebrafish were housed in a light- and temperature-controlled aquaculture facility with a standard 14:10 h day/night photoperiod and fed with live brine shrimp twice a day and dry flake once a day. Four to five pairs of them were set up for each mating, and about 200–300 embryos can thereby be generated. Embryos were maintained at 28 °C in fish water (0.2% Instant Ocean Salt in deionized water, pH 6.9–7.2, conductivity 480–510 μS/cm and hardness 53.7~71.6 mg/L CaCO_3_). Embryos were washed and staged at 6 and 24 hpf (hours post-fertilization). The zebrafish facility at Hunter Biotechnology, Inc., is accredited by the Association for Assessment and Accreditation of Laboratory Animal Care (AAA LAC) International.

### 4.3. Chemical Treatment

Larval zebrafish (72 hdf) were grouped (30 fishes per group) and distributed into 6-well plates (Nest Biotech, China) in 3 mL fresh fish water for a 4-h treatment. Zebrafish treated with fish water and with 0.9% NaCl solution were used as the blank control (untreated) and the vehicle control, respectively. After yolk sac microinjection with SA (10 nL each), zebrafish were subject to visual observation and image acquisition under a dissecting stereomicroscope (Olympus Ltd, Tokyo, Japan).

### 4.4. Determination of MNLC and LC_10_

The maximum non-lethal concentration (MNLC) and 10% lethal concentration (LC_10_) of SA were determined using zebrafish larvae from 72 to 120 hpf. Mortality was recorded every 24 h, and dead zebrafish were defined as the absence of heartbeat under the dissecting stereomicroscope. Eight concentrations of SA (0.1, 0.5, 1.0, 1.5, 2.0, 2.5, 5.0, 10.0 μg/mL) were used according to the preliminary tests. The mortality curve was generated using Origin 8.0 (OriginLab, Northampton, MA, USA). MNLC and LC_10_ were determined with logistic regression.

### 4.5. Cardiovascular Toxicity Assessment

Four dosages at 1/10 MNLC (0.15 μg/mL), 1/3 MNLC (0.5 μg/mL), MNLC (1.5 μg/mL) and LC_10_ (2.0 μg/mL) were selected for assessing cardiovascular toxicity of SA. After the treatment on zebrafish laevae from 72 to 120 hpf, 10 zebrafish from each group were randomly selected for visual observation and imaging acquisition of specific phenotypic endpoints under the dissecting stereomicroscope. The occurrence of pericardial edema, abnormal circulation (decrease and absence), thrombosis and hemorrhage were thereby evaluated by double-blind observation and the heart rate (atrial rate and ventricular rate) and heart rhythm counted, as described by our previous study [34].

### 4.6. Statistical Analysis

All tests were replicated until the experimental condition was optimized. Sigmoidal regression for the concentration-response curve was used for estimation of MNLC and LC_10_ in Origin 8.0. The heart rate and heart rhythm were expressed as the mean ± standard error (SE). One-way ANOVA followed by Dunnett’s test were used to compare differences between different groups. All statistical analyses were performed using SPSS 16.0 software (SPSS Inc., Chicago, IL, USA).

## 5. Conclusions

SA has been a well-developed and widely-applied agent for many therapeutic purposes, with no doubt of its safety. However, by using a zebrafish cardiotoxicity assay, this study revealed that exposure to SA at only the 1/3 MNLC dose was sufficient to induce adverse events in the cardiovascular system, including malformations and malfunctions. Therefore, from this moment on, such cardiotoxicity of SA should be clinically considered and further confirmed before it causes actual irreparable harm to humans. Furthermore, in practical applications, more attention should be given to its dose reduction and treatment-course shortening. This conventional zebrafish cardiotoxicity assay is predictive, sensitive, easily available and inexpensive with a short testing time and could occupy an important niche between traditional representative animal models and *in vitro* systems, not only for validating and describing known toxicities, but also for detecting the unforeseen during drug research and development.
